# The right atrium value in patients operated for tetralogy of Fallot

**DOI:** 10.1186/1532-429X-17-S1-Q104

**Published:** 2015-02-03

**Authors:** Gianluca Trocchio, Lamia Ait-Ali, Nicola Stagnaro, Francesca Rizzo, Maurizio Marasini, Pierluigi Festa

**Affiliations:** 1 Pediatric Cardiology, Fondazione G. Monasterio CNR-Regione Toscana, Ospedale del cuore, Massa, Italy; 2 Cardiovascular Department, Pediatric Cardiology, Giannina Gaslini Institute, Genova, Italy; 3 Radiology Unit, Giannina Gaslini Institute, Genova, Italy; 4 Istituto di Fisiologia Clinica, CNR,, Massa-Pisa, Italy

## Background

Left atrium enlargement is directly proportional to the severity of underlying clinical (or subclinical) cardiovascular disease. Right atrium (RA) measurements are not routinely performed despite their prognostic value has been demonstrated in chronic heart failure, atrial and pulmonary arterial hypertension. The aim of the study was to evaluate RA dimensions and volumes by cardiac Magnetic Resonance (CMR) in a large cohort of operated Fallot (opTF) compared to a control group, and correlate the RA size to traditional prognostic parameters and clinical events.

## Methods

From 2004 to 2013, 370 consecutive opTF referred to 2 cardiac centers have been evaluated by means of a protocol comprehensive of detailed clinical and surgical history, ECG, trans-thoracic Echo, CMR, cardio-pulmonary exercise test. Exclusion criteria were: age <10 yrs, contraindication to CMR, associated major cardiac anomalies. The final study population included 272 opTF (208 males; mean age 24.5±11 yrs, range 10-63). Median age at primary repair was 2 yrs; 46 (13%) patients had been re-operated for right ventricle (RV) outflow tract reconstruction. Follow-up from primary repair was 21±8.7 yrs. The control group was constituted by 79 age and sex matched healthy volunteers. The RA was measured by tracing and summing the maximum RA linear dimensions lying on the main 3 axis (antero-posterior, supero-inferior, latero-lateral) from an ECG-gated volumetric acquisition triggered in diastole. For the last 71 patients RA volumes were also assessed.

## Results

RA diameters resulted significantly higher in opTF compared to control group (165.6±29 mm vs 147±18.5 mm, p<0.001), even if patients with more than mild tricuspid regurgitation were excluded (161.5±25.5 mm, p<0.001). RA diameters correlated with age at primary repair (Pearson R:0.36, p<0.001), QRS length at ECG (Pearson R:0.37, p<0.001), RV end-diastolic and end-systolic volumes (Pearson R:0.45, p<0.001 and Pearson R:0.44, p<0.001, respectively), RV mass (Pearson R:0.27, p<0.001), late gadolinium enhancement score (Pearson R:0.25, p<0.001), and left ventricle (LV) end-diastolic and end-systolic volumes (Pearson R:0.28, p<0.001 and Pearson R:0.26, p<0.001, respectively). Moreover, RA diameters inversely correlate to RV ejection fraction (EF) (Pearson R:-0.31, p<0.001), LV EF (Pearson R:-0.17, p=0.007), and peak VO2 (Pearson R:-0.27, p<0.001), while no correlation was found with pulmonary regurgitation (Pearson R:-0.05, p=0.37) and RV systolic pressure (Pearson R:0.08, p=0.2). Finally, RA diameters and volumes resulted to be independent factors for major atrial arrhythmias.

## Conclusions

RA is dilated in opTF independently of tricuspid regurgitation. RA dimensions are inversely related to functional capacity, RV and LV function. RA enlargment is associated to occurrence of atrial arrhythmias. Further studies are required to brighten the prognostic value of RA in opTF.

## Funding

N/A.

